# Extracellular vesicles from adipose-derived stem cells promote microglia M2 polarization and neurological recovery in a mouse model of transient middle cerebral artery occlusion

**DOI:** 10.1186/s13287-021-02668-0

**Published:** 2022-01-20

**Authors:** Xiaowen Hu, Jiaji Pan, Yongfang Li, Yixu Jiang, Haoran Zheng, Rubing Shi, Qi Zhang, Chang Liu, Hengli Tian, Zhijun Zhang, Yaohui Tang, Guo-Yuan Yang, Yongting Wang

**Affiliations:** 1 Shanghai Sixth People’s Hospital and School of Biomedical Engineering, Shanghai Jiao Tong University, Shanghai, 200030 China; 2 Department of Rehabilitation and Institute of Neurology, Ruijin Hospital, School of Medicine, Shanghai Jiao Tong University, Shanghai, 200025 China

**Keywords:** Adipose-derived stem cell, Cerebral ischemia, Extracellular vesicles, Microglial polarization, miRNAs

## Abstract

**Background:**

Adipose-derived stem cells (ADSCs) and their extracellular vesicles (EVs) have therapeutic potential in ischemic brain injury, but the underlying mechanism is poorly understood. The current study aimed to explore the contribution of miRNAs in ADSC-EVs to the treatment of cerebral ischemia.

**Methods:**

After the intravenous injection of ADSC-EVs, therapeutic efficacy was evaluated by neurobehavioral tests and brain atrophy volume. The polarization of microglia was assessed by immunostaining and qPCR. We further performed miRNA sequencing of ADSC-EVs and analyzed the relationship between the upregulated miRNAs in ADSC-EVs and microglial polarization-related proteins using Ingenuity Pathway Analysis (IPA).

**Results:**

The results showed that ADSC-EVs reduced brain atrophy volume, improved neuromotor and cognitive functions after mouse ischemic stroke. The loss of oligodendrocytes was attenuated after ADSC-EVs injection. The number of blood vessels, as well as newly proliferated endothelial cells in the peri-ischemia area were higher in the ADSC-EVs treated group than that in the PBS group. In addition, ADSC-EVs regulated the polarization of microglia, resulting in increased repair-promoting M2 phenotype and decreased pro-inflammatory M1 phenotype. Finally, STAT1 and PTEN were highlighted as two downstream targets of up-regulated miRNAs in ADSC-EVs among 85 microglia/macrophage polarization related proteins by IPA. The inhibition of STAT1 and PTEN by ADSC-EVs were confirmed in cultured microglia.

**Conclusions:**

In summary, ADSC-EVs reduced ischemic brain injury, which was associated with the regulation of microglial polarization. miRNAs in ADSC-EVs partly contributed to their function in regulating microglial polarization by targeting PTEN and STAT1.

**Supplementary Information:**

The online version contains supplementary material available at 10.1186/s13287-021-02668-0.

## Background

Stroke is the second leading cause of death and the major cause of disability worldwide. About 80% of stroke cases are ischemic stroke characterized by thromboembolic occlusion of the cerebral artery [1, 2]. Current clinical therapeutic approaches mainly focus on removing the blockage by thrombolysis or thrombectomy [3, 4]. However, these treatments usually have a narrow therapeutic window [3]. Therefore, new therapeutic strategy with wider treatment window is needed.

Microglia are main immune cells in the brain and play a critical role in modulating the microenvironment of the central nervous system (CNS). Microglia are activated in response to ischemic stroke [5]. In the early phase of acute brain ischemia, an anti-inflammatory M2 microglia phenotype increases from 1 to 3 days and peaks by 3 to 5 days post ischemic brain injury. In the same phase, a pro-inflammatory M1 microglia phenotype gradually increases over time from day 3 onward and maintain elevated for at least 14 days after ischemic stroke [6]. It has been well documented that M1 microglia secrets various pro-inflammatory cytokines, such as interleukin-1β (IL-1β), interleukin-23 (IL-23), tumor necrosis factor-α (TNF-α), and inducible nitric oxide synthase (iNOS), thus aggravates the neurological deficits [7, 8]. The activation of A1 reactive astrocytes is closely related to the release of IL-1α, TNF-α and C1q by classical activated microglia. A1 astrocytes further release inflammatory factors and destroy adjacent neurons and oligodendrocytes [9]. In contrast, M2 microglia promote repair and anti-inflammatory reactions [10, 11]. Previous studies have demonstrated that M2 microglia derived exosomes can reduce apoptosis of neurons and glial scar formation after cerebral ischemia [12, 13]. Arg-1 high-expressing M2 microglia promote the proliferation and differentiation of oligodendrocyte progenitor cells (OPCs) [9]. In summary, the dynamic polarization of microglia and their interaction with neurons, astrocytes, and oligodendrocytes play important roles in the pathological process of cerebral ischemia. Shifting microglial polarization to M2 phenotype has been proposed to be a new therapeutic strategy for cerebral ischemia [14, 15].

Previous studies have demonstrated that the administration of mesenchymal stem cells (MSCs) and their derived extracellular vesicles (EVs) can substantially improve the neurological deficits after ischemic stroke [16, 17]. Compared to other sources, adipose-derived stem cells (ADSCs) have the advantages of being abundant and easy to obtain [18]. Since their discovery in 2001, the therapeutic potential of ADSCs has been explored in wound healing, multiple sclerosis and various ischemic diseases. For ischemic stroke, ADSCs have been indicated to be beneficial by reducing apoptosis, the release of inflammatory factors, glial scar formation, white matter injury and promoting angiogenesis, the migration and differentiation of endogenous neural stem cells, synaptic remodeling [19–23]. However, the survival rate of ADSCs after transplantation is very low, so it is inferred that ADSCs achieve these therapeutic effects mainly through paracrine mechanisms [21, 23].

EVs are important carriers that facilitate cell–cell communication. They contain a large number of biologically functional proteins and miRNA molecules [24]. Administration of ADSC-EVs can not only achieve comparable therapeutic effect of ADSCs, but also avoid the risks and shortcomings of stem cell transplantation such as concerns of tumorgenicity. Several studies have shown that ADSCs and their derived cell-free conditioned medium, extractions or EVs can promote M2 polarization of macrophage [25, 26]. It has been reported that ADSCs or ADSC-EVs transplantation improved myocardial injury by promoting M2 polarization of macrophages in myocardial ischemia models [27, 28]. In obese mice, ADSCs up-regulate the expression of Arg-1 by carrying active STAT3 to induce the anti-inflammatory M2 phenotype of macrophages, thereby reducing inflammation and promoting metabolic homeostasis [29]. However, the effects of ADSC-EVs on microglial polarization in the brain have not been demonstrated. In the present study, we aim to investigate whether ADSC-EVs regulate the polarization of microglia and contribute to neurological recovery after ischemic stroke. Among different cargos contained in the EVs, miRNAs are the most explored and have been shown to be involved in many physiological and pathological processes [30]. Therefore, we hypothesize that ADSC-EVs exerts regulatory functions at least in part by delivering miRNAs to recipient cells.

## Methods

### Isolation and characterization of ADSCs

ADSCs were isolated from the inguinal subcutaneous adipose tissue of 6-week-old male ICR mice. The dissected adipose tissue was digested with 0.1% type II collagenase (Sigma-Aldrich, Saint Louis, MO) at 37 °C for 30 min under 20 rpm shaking conditions. Enzymatic activity was neutralized with an equal volume of low glucose Dulbecco’s modified Eagle medium (L-DMEM, HyClone, Logan, UT) containing 10% fetal bovine serum (Gibco, Carlsbad, CA). After filtration through a 70 μm filter and centrifugation at 300 g for 5 min, cells were resuspended and seeded in complete medium at 37 °C with 5% carbon dioxide and saturated humidity. The complete medium was composed of L-DMEM with 10% FBS and 1% penicillin/streptomycin (Invitrogen, Carlsbad, CA) and replaced every 2 or 3 days. Cells were harvested with 0.25% trypsin–EDTA, and all experiments were performed using cells between passage 2 and 5. Surface markers of ADSCs were validated by flow cytometry (Accuri C6, BD, Franklin Lakes, NJ). Briefly, the single-cell suspension was washed twice with PBS containing 0.5% BSA and then incubated in dark for 30 min at 4 °C with each of the following antibodies directed against murine antigens: anti-CD29-PE (Cat#562801, BD), anti- CD34-Alexa Flour 647(Cat#560233, BD), anti-CD44-APC (Cat#561862, BD), and anti-CD45-PerCP (Cat#561047, BD). Cell suspension without antibodies was used as control. Adipogenic differentiation complete medium (Cyagen, Guangzhou, China) and osteogenic differentiation complete medium (Cyagen) were used to verify the multiple differentiation capabilities of ADSCs.

### EVs isolation, identification and labeling

EVs were purified from ADSCs supernatant by ultracentrifugation following a published protocol [31]. The supernatant was collected after 48 h of culture and passed through a 0.22 μm filter before ultracentrifugation. The size distribution of ADSC-EVs was evaluated by nanoparticle tracking analysis (NTA, Brookhaven, New York, NY). Transmission electron microscopy (TEM, Thermo Scientific, Waltham, MA) was used to identify the morphological characteristics of ADSC-EVs. ADSCs lysate and ADSC-EVs were analyzed for the expression of exosomal markers by Western blot with the following antibodies: anti-CD63 (Santa Cruz Biotechnology, Santa Cruz, CA), tumor susceptibility gene 101 (TSG101, Abcam, Cambridge, UK) and β-actin (Abcam). Labeling of ADSC-EVs was performed with PKH26 red fluorescent cell linker kit (Sigma-Aldrich) according to the manufacturer’s instructions.

### The mice model of transient middle cerebral artery occlusion (tMCAO)

Adult male ICR mice (25–30 g) were anesthetized with 1.5–2% isoflurane and 30%/70% oxygen/nitrous oxide. tMCAO was performed as previously described [32, 33]. Briefly, the left common carotid artery, internal carotid artery and external carotid artery were separated and ligated temporarily. An incision was made between the two ligations on external carotid artery. Then a 6-0 nylon suture coated with silica gel was inserted through the incision into the ipsilateral middle cerebral artery. A laser Doppler flowmetry (Moor Instruments, Devon, UK) was used to determine the successful occlusion of middle cerebral artery by monitoring the decrease of surface cerebral blood flow (CBF) to 10% of its baseline. Reperfusion was performed by withdrawing the suture 1.5 h after the occlusion and the reperfusion was confirmed by the recovery of surface CBF to 70% of baseline. In the sham group, an identical surgical procedure was performed without ligation of any arteries. A laser speckle imaging system (RWD Life Science, Shenzhen, China) was used to record and display the changes of cerebral blood flow in the ischemic zone of tMCAO mice.

### ADSC-EVs administration

After tMCAO, animals were randomly divided into the PBS group and ADSC-EV group (*n* = 14). Animals in the ADSC-EV group received daily injections of ADSC-EVs through tail vein during 1–7 days after tMCAO (100 μg ADSC-EVs in 200 μl PBS per day); while animals in the PBS group were injected with 200 μl PBS per day. No injection was performed in the sham group (*n* = 4). To verify the uptake of ADSC-EVs by brain microglia in vivo, PKH-26 labeled ADSC-EVs (100 μg) were intravenously injected to the mouse at 1 day after tMCAO. Mice were sacrificed 1 h after the injection, and then Iba1 immunostaining was performed.

### Neurobehavioral tests

The modified neurological severity score (mNSS) was used to evaluate the neurobehavioral function of the animals at 1 to 14 days after tMCAO. Before surgery, the animals were subjected to a 3 days training on the rotarod. Then, rotarod baseline data was collected before surgery. After surgery, rotarod test was carried out at 3, 7, and 14 days after tMCAO. Hanging wire test was performed at 14 days after tMCAO [34]. Mice were subjected to a 180 s hanging on a suspended wire. One point was subtracted from the initial score of 10 for each fall during the test. The average score of each group was presented as a Kaplan–Meier-like curve. Step through test and T-maze were used to detect spatial working memory and cognition function at 14 days after tMCAO. In T-maze spontaneous alternation experiment, normal mice tended to choose different goal arms every time, while neurologically impaired mice presented lower alternation rate in 10 trials. In the step through test, the training was performed using the smart cage system where electrical stimulation was delivered when the animal entered the dark zone. After 24 h, mice were placed back to the smart cage again and their continuous moving trace within 10 min were recorded. The total time spent in the dark zone and entries from light zone to dark zone were calculated. Mice with impaired memories showed the inclination to enter and stay in the dark zone.

### Atrophy volume assessment

After transcardial perfusion with PBS and 4% paraformaldehyde (PFA, Sinopharm Chemical Reagent, Shanghai, China), the mouse brain was removed and placed in 4% PFA overnight before transferred into 30% sucrose for cryoprotection. A series of 30-μm-thick brain sections were collected, among which 7 sections spaced 300 μm apart were stained with cresyl violet solution. Atrophy area (Δ*S*_*n*_) was determined by calculating the staining area loss in the ipsilateral compared to contralateral hemispheres in ImageJ. The brain atrophy volume was calculated using the following formula: *V* = Σ *h*/3[Δ*S*_*n*_ + (Δ*S*_n_*Δ*S*_*n*+1_)1/2 + Δ*S*_*n*+1_], h represented the distance between the two adjacent brain sections.

### Immunofluorescence staining

The brain sections were successively treated with 0.1% TritonX-100 for 15 min, 10% BSA for 1 h at room temperature, and then incubated overnight at 4 °C with the following antibodies: anti-CD16/32 (BD)/anti-Iba1 (WAKO, Osaka, Japan), anti-Arg-1 (Santa cruz)/ anti-Iba1 (WAKO), anti-MBP (Abcam), anti-CD31 (R&D), anti-CD31 (R&D)/anti-Ki67 (Abcam). After washing with PBS for 3 times, the sections were incubated with different fluorophores-conjugated secondary antibodies (Invitrogen) for 1 h at room temperature. After washing with PBS for 3 times, the brain sections were mounted with DAPI-containing antifade mounting medium (Invitrogen). Three brain sections from each mouse were imaged using a FV10i confocal microscope (Olympus, Tokyo, Japan). All settings were kept constant during picture acquisition. The mean fluorescence integrated intensities and mean area were calculated by setting the accordingly scale bar in Image J.

### RNA extraction and real-time PCR

Total RNA from ADSCs was extracted using TRIzol reagent (Invitrogen) according to the manufacturer’s protocol. miRNA-containing total RNA extraction was conducted by miRNeasy serum/plasma advanced kit (QIAGEN, Hilden, Germany) according to the manufacturer’s protocol. cDNA synthesis and real time PCR of miRNA were performed by miRcute plus miRNA cDNA synthesis kit and miRcute plus miRNA qPCR kit (SYBR Green, Tiangen Biotech, Beijing, China). The expression of mRNA or miRNA was tested by a fast real-time PCR system (7900 HT, ABI, Foster City, CA). GPADH was used as the endogenous control of mRNA. The relative expression was normalized to that in the control group. The sequences of the primers used in this study were listed in Table 1.

**Table 1:** Primers for mRNA real-time polymerase chain reaction

Gene		Primer
GAPDH	Forward	5′-AGGTCGGTGTGAACGGATTTG-3′
	Reverse	5′-TGTAGACCATGTAGTTGAGGTCA-3′
Arg-1	Forward	5′-GTGAAGAACCCACGGTCTGT-3′
	Reverse	5′-GCCAGAGATGCTTCCAACTG-3′
CD16	Forward	5′-TTTGGACACCCAGATGTTTCAG-3′
	Reverse	5′-GTCTTCCTTGAGCACCTGGATC-3′
IL-1β	Forward	5′-CCAGCTTCAAATCTCACAGCAG-3′
	Reverse	5′-CTTCTTTGGGTATTGCTTGGGATC-3′
TNF-α	Forward	5′-CCCTCACACTCAGATCATCTTCT-3′
	Reverse	5′-GCTACGACGTGGGCTACAG-3′
iNOS	Forward	5′-CAAGCACCTTGGAAGAGGAG-3′
	Reverse	5′-AAGGCCAAACACAGCATACC-3′
PTEN	Forward	5′-TGGATTCGACTTAGACTTGACCT-3′
	Reverse	5′-GCGGTGTCATAATGTCTCTCAG-3′
STAT1	Forward	5′-GGAAGGGGCCATCACATTCA-3′
	Reverse	5′-TGTAGGGCTCAACAGCATGG-3′

### Western blot analysis

The concentrations of protein samples were determined by BCA protein assay (Pierce, Rockford, IL). The primary antibodies were anti-CD63 (SC-15363, 1:1000, Santa Cruz), anti-TSG101 (Ab83, 1:500, Abcam), anti β-actin (Proteintech 66009, 1:2000, Wuhan, China).

### Culture of primary microglia

Mixed glial cultures were prepared from cerebral cortices of 1‐day‐old SD rats according to the previous method [35]. After 0.25% trypsin digestion for 10 min, cortical cells were plated in DMEM with 10% FBS and 1% penicillin/streptomycin and cultured at 37 °C in 5% CO2 incubator with saturated humidity. Medium was changed every 3 days. Ten days after plating, we separated microglia from mixed glia cells by shaking at 200 rpm, 37 °C for 30 min. Then the microglia were seeded in DMEM with 10% FBS at a density of 300,000 cells/ml.

### In vitro cellular uptake of ADSC-EVs and microglial polarization studies

PKH-26 labeled ADSC-EVs were co-incubated with primary microglia for 6 h, and then Iba1 immunofluorescence staining were performed to verify the cellular uptake of ADSC-EVs by cultured microglia in vitro. ADSC-EVs treatment of primary microglia was performed under normal culture, lipopolysaccharides (LPS) induction, and oxygen–glucose deprivation (OGD) injury conditions. Under normal condition, M1 and M2 marker of microglia were detected 24 h after ADSC-EVs administration (0, 20, 40 μg/ml). For LPS induction, primary microglia were subjected to 50 ng/ml LPS for 6 h after ADSC-EVs pretreatment (0, 20, 40 μg/ml) for 24 h. The expression of Arg-1, CD16, IL1β, TNF-α and iNOS in microglia were detected by qPCR. After 1 h of OGD, primary microglia were subjected to normal oxygen level and treated with ADSC-EVs (0, 20, 40 μg/ml) for 24 h. The expression of Arg-1 and CD16 in microglia was detected by qPCR.

### miRNA sequencing and IPA analysis

The miRNA sequencing of ADSC-EVs was performed using fibroblast-EVs as a control (Novelbio, Shanghai, China). DESeq2 algorithm was applied to filter the differentially expressed genes [36]. *p* value and FDR analysis were subjected to the following criteria: fold change > 1.5 or < 0.667, and *p* value < 0.05, FDR < 0.05. The heat-map was plotted using MeV software. The scale represented signal intensity of log2 (TPM) values. The relationships between up-regulated miRNAs and microglial polarization-related proteins were detected by IPA analysis with selecting miRecord, Tarbase, TargetScan in miroRNA-mRNA interactions and miRBase in additional sources. U6 was used as the control in miRNA qPCR. The sequences of the primers used in this study were listed in Table 2.

**Table 2:** Primers for miRNA real-time polymerase chain reaction

miRNA	Primer
mmu-miR-93-3p	CCACTGCTGAGCTAGCACTTCCCG
mmu-miR-106b-5p	CGCGCTAAAGTGCTGACAGTGCAGAT
mmu-miR-128-3p	CGCCGTCACAGTGAACCGGTCTCTTT
mmu-miR-144-3p	GCGGCGCGCGTACAGTATAGATGATG
mmu-miR-146a-5p	GCGCGTGAGAACTGAATTCCATGGGT
mmu-miR-200a-3p	GCCGCGCTAACACTGTCTGGTAACGAT
mmu-miR-200b-3p	GCCGCGCGTAATACTGCCTGGTAATGA
mmu-miR-200c-3p	GCGCGTAATACTGCCGGGTAATGATGG
mmu-miR-223-3p	GCGCGCTGTCAGTTTGTCAAATACCCC
mmu-miR-345-5p	CGCTGACCCCTAGTCCAGTGCTT
mmu-miR-363-3p	CGCGCGAATTGCACGGTATCCATCTGT
mmu-miR-376a-3p	GCCGCGATCGTAGAGGAAAATCCACGT
mmu-miR-429-3p	GCGCGCTAATACTGTCTGGTAATGCCG
mmu-miR-451a	GCGCGCGAAACCGTTACCATTACTGA

### miRNA mimic infection of BV_2_ microglia

BV_2_ microglia were transfected with 100 nM negative control, or 100 nM miR-93-3p mimics, miR-128-3p mimics, miR-144-3p mimics, miR-146a-5p mimics, miR-223-3p mimics, miR-106b-5p mimics, miR-200a-3p mimics, miR-200b-3p mimics, or miR-363-3p mimics, separately. Twenty-four hours after transfection, the expression of STAT1 and PTEN were detected by qPCR using GAPDH as the internal standard. The sequences of the primers used in this study were listed in Table 1.

### Statistical analysis

All data were presented as means ± standard error of mean (SEM). Two-tailed t-test was used to compare the means of two groups. One-way ANOVA analysis with Turkey multiple-comparisons posttest was used to analyze the significant difference of multiple groups. *p* value was calculated by GraphPad Prism 8.0 software and *p* < 0.05 was considered to be statistically significant.

## Results

### Isolation and identification of ADSC-EVs

**Fig. 1:**
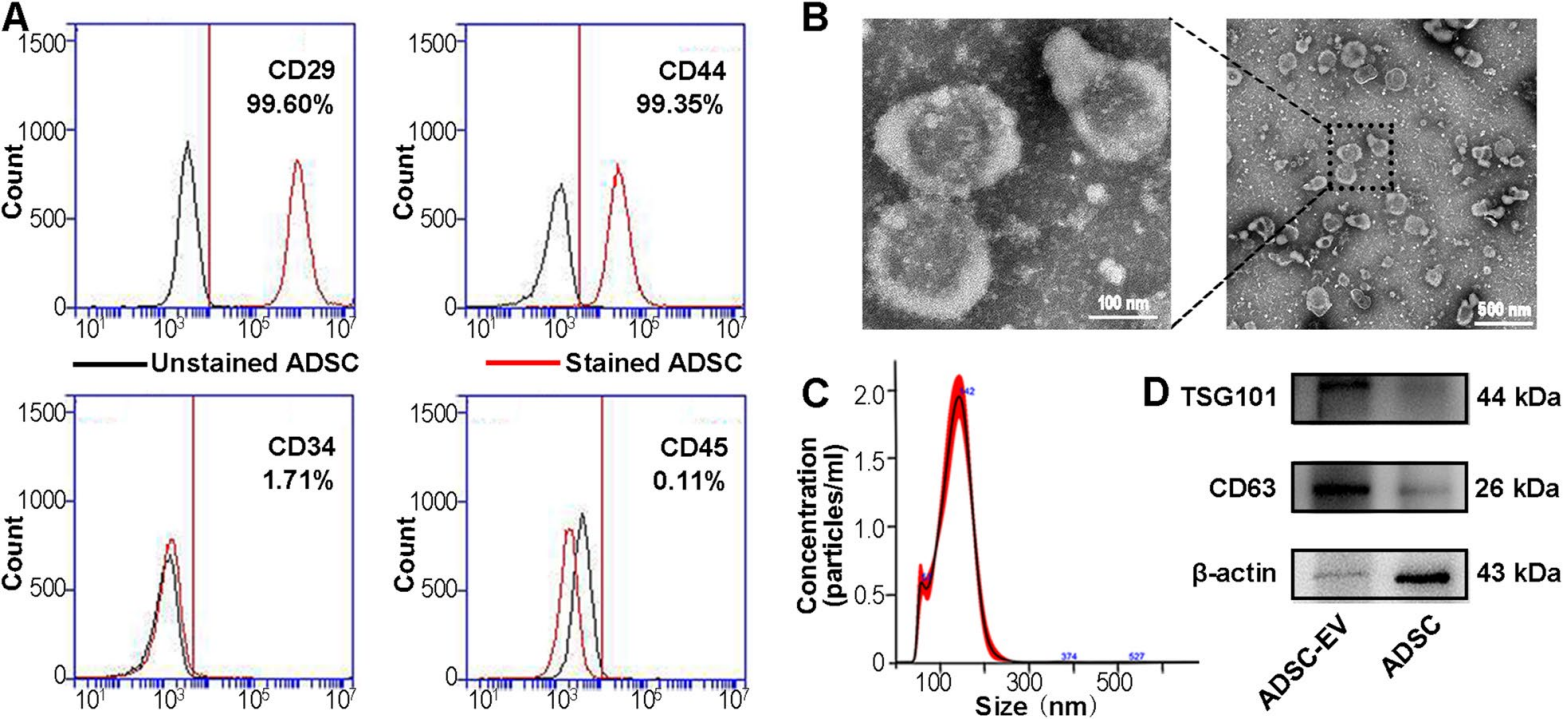
Characterization of ADSCs and ADSC-derived EVs. **A** Flow cytometric analysis of ADSCs surface markers CD29 and CD44, and negative markers CD34 and CD45. Black curve: Unstained ADSCs; Red curve: ADSCs stained by fluorescent labeled primary antibodies. **B** Representative TEM image of ADSC-EVs. Scale bar = 500 nm. The enlarged picture showed the clear structure of ADSC-EVs. Scale bar = 100 nm. **C** Size distribution of ADSC-EVs measured by NTA. **D** Expressions of the EV markers CD63, TSG101, and β-actin confirmed by Western blot. ADSC-EVs lysate was loaded into the left lane and ADSCs lysate was loaded into the right lane

To obtain allogeneic ADSC-EVs, we first obtained subcutaneous white fat tissue (WAT) from the bilateral inguinal region of 6-week-old ICR male mice and isolated the primary ADSCs according to a method described previously [37]. Following the criteria for identifying ADSCs [38], we detected the expression of each cluster of differentiation (CD) marker by flow cytometry. The result showed that after second passage (P2), 99.6% of the ADSCs were CD29^+^, 99.3% were CD44^+^, 1.7% were CD34^+^, and 0.1% were CD45^+^, which was consistent with the identification criteria for ADSCs (Fig. 1A). Cell morphology of ADSCs were shown in Additional file 1: Fig. S1A. Oil Red O staining and Alizarin Red S staining confirmed the adipogenic and osteogenic differentiation of ADSCs, respectively (Additional file 1: Fig. S1B-C). In addition, flow cytometric analysis of P3-P5 generation ADSCs also exhibited similar surface marker properties. Given these results and the gradually diminished proliferation ability of cultured ADSCs, we extracted EVs from the condition medium of P2-P5 primary ADSCs by ultracentrifugation. As shown in Fig. 1B, C, the mean diameter of ADSC-EVs particles was 142 ± 39.4 nm and a typical cup-shaped morphology was presented by TEM. Western blot analysis revealed the abundance of two EV markers CD63 and TSG101 (Fig. 1D). All these data indicated that ADSC-EVs were successfully isolated.

### ADSC-EVs administration reduced brain atrophy and improved neuromotor and cognitive recovery after ischemic stroke in mice

**Fig. 2:**
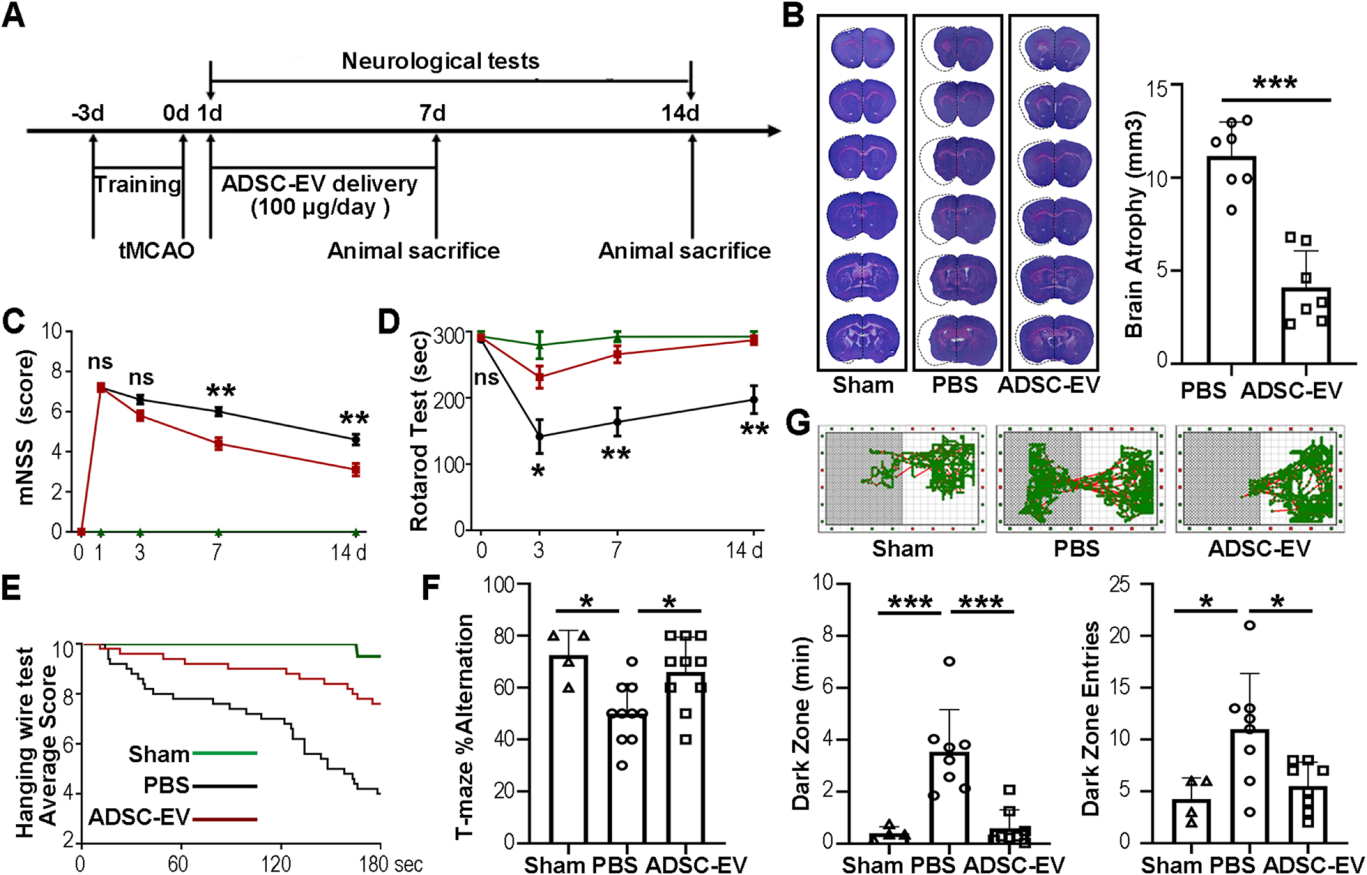
ADSC-EVs reduced brain atrophy volume and promoted neural functional recovery. **A** Experimental schedule. ADSC-EVs injection was performed daily during 1–7 days after tMCAO. **B** Representative photomicrographs of brain coronal sections stained with cresyl violet and quantification of brain atrophy. The white dotted line showed the mirror image of the contralateral hemisphere. *n* = 7. **C** The results of mNSS evaluation before tMCAO and 1, 3, 7, and 14 days after the operation. *n* = 4 in the sham group, *n* = 10 in the PBS and ADSC-EVs groups. **D** Rotarod test before tMCAO and 3, 7, and 14 days after the operation. *n* = 4 in the sham group, *n* = 10 in the PBS and ADSC-EVs groups. **E** Average fall score of mice during a 180-s hanging wire test at 14 days after tMCAO. *n* = 4 in the sham group, *n* = 10 in the PBS and ADSC-EVs groups. Green curve: Sham group; Black curve: PBS group; Red curve: ADSC-EVs group. **F** T-maze test showed the ratio of spontaneous alternation at 14 days after tMCAO. *n* = 4 in the sham group, *n* = 10 in the PBS and ADSC-EVs groups. **G** Representative moving trace of mice recorded in step through test at 14 days after tMCAO. Bar graphs at the bottom showed the quantified total time spent in the dark zone and the number of dark zone entries of each mouse. *n* = 4 in the sham group, *n* = 8 in the PBS and ADSC-EVs groups. Data were presented as mean ± SEM, *p < 0.05, **p < 0.01, and ***p < 0.001, ns = not significant

To investigate the therapeutic effects of ADSC-EVs on cerebral ischemia in mice, we designed experimental schedule as shown in Fig. 2A. Laser speckle imagines showed the changes of cerebral blood flow in tMCAO mice during occlusion and after reperfusion (Additional file 1:Fig. S2). Cresyl violet staining at 14 days after cerebral ischemia showed that ADSC-EVs treatment decreased the brain atrophy volume compared to the PBS group (*p* < 0.001, Fig. 2B). To verify the effect of ADSC-EVs treatment on neurological function recovery after tMCAO, we performed neurobehavioral tests. The mNSS showed that the neurological deficts in the ADSC-EV group was less than that in the PBS group at 7 and 14 days after tMCAO (*p* < 0.01, Fig. 2C). Rotarod test showed that the mice in the ADSC-EV group spent more time on the rotarod at 3, 7, and 14 days after tMCAO (*p* < 0.05, Fig. 2D). In the hanging wire test, the average score of mice in the ADSC-EV group was higher than that in the PBS group (Fig. 2E), indicating that ADSC-EVs treatment could improve the neuromotor and coordination functions of mice after cerebral ischemia. T-maze alternation experiments were performed at 14 days after tMCAO. The alternation rate of mice in the PBS group was significantly lower than that in the sham group (*p* < 0.05), while ADSC-EVs treatment increased the alternation rate compared to the PBS group (*p* < 0.05, Fig. 2F). The moving traces of mice in step through test were recorded using a smart cage system at 14 days after tMCAO. The time spent in dark zone and the number of entering to the dark area were increased after cerebral ischemic injury compared to the sham group (*p* < 0.05), while ADSCs reduced both the time spent and the number of entering into the dark area compared to the PBS group (*p* < 0.05, Fig. 2G). The results of T-maze and Step through tests indicated that ADSC-EVs treatment improved cognitive functions of mice after cerebral ischemia.

### ADSC-EVs protected against white matter injury after mouse cerebral ischemia

**Fig. 3:**
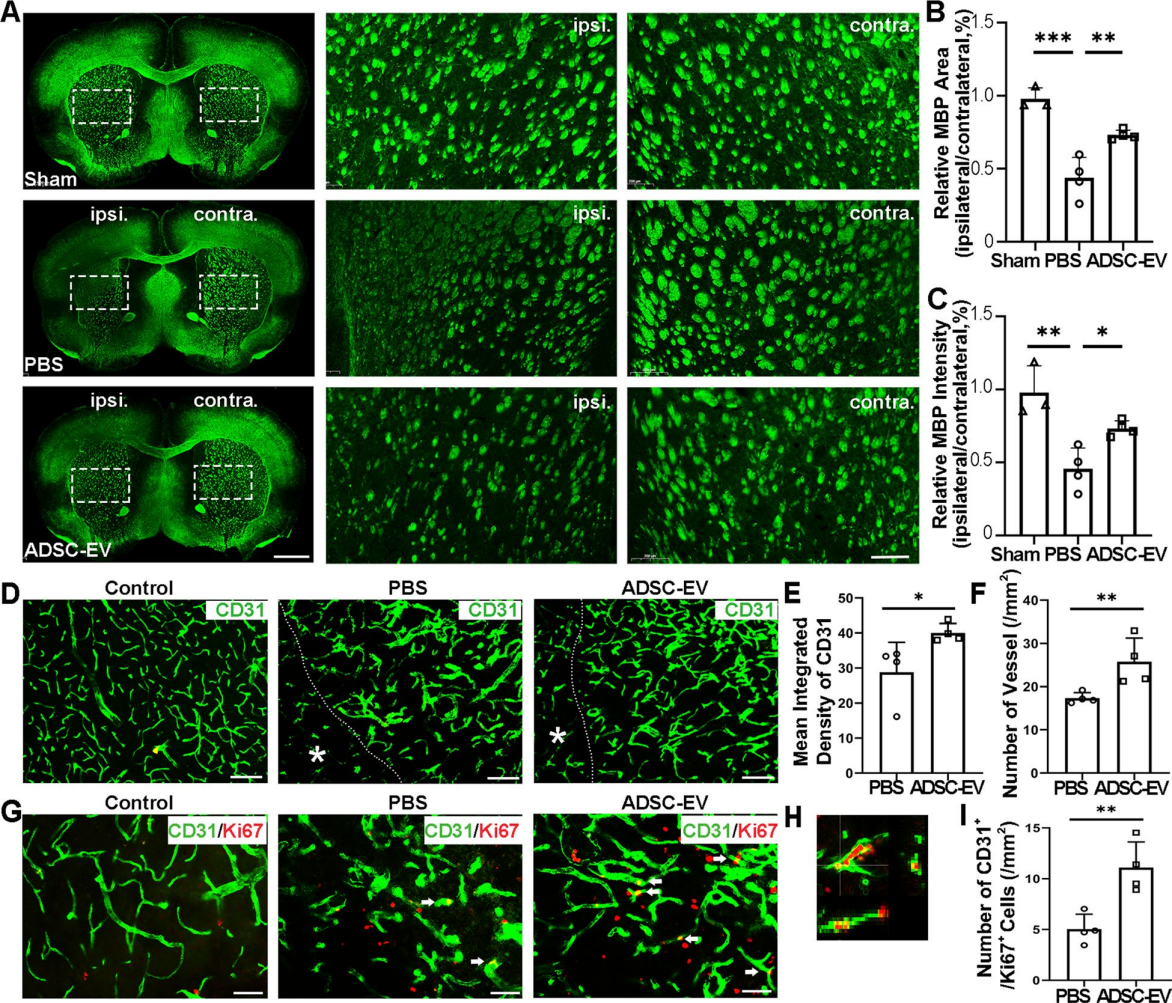
ADSC-EVs attenuated white matter injury and promoted angiogenesis in tMCAO mice. **A** Immunofluorescence staining of MBP at 14 days after tMCAO in the PBS and ADSC-EVs groups. Scale bar = 1000 μm. The enlarged pictures of white square revealed MBP staining in the striatum of the ipsilateral and the contralateral hemisphere. Scale bar = 200 μm. **B** Quantitative analyses of the ratio of mean MBP area in ipsilateral to contralateral striatum. **C** Quantitative analyses of the ratio of mean MBP intensity in ipsilateral to contralateral striatum. *n* = 3 in the sham group, *n* = 4 in the PBS and ADSC-EVs groups. **D** Immunofluorescence staining of CD31^+^ microvessels in the ipsilateral hemisphere of mice brain after tMCAO in the PBS and ADSC-EVs groups. The images of the contralateral hemisphere were presented as control. White star indicated the core area of infarction and the white dashed line represented the boundary between the ischemic core area and peripheral area. Scale bar = 50 μm. **E** Mean integrated density of CD31^+^ microvessels in the perifocal region at 14 days after tMCAO. **F** Quantifications of CD31^+^ microvessels numbers in the perifocal region at 14 days after tMCAO. *n* = 4 in the PBS and ADSC-EVs groups. **G** Immunofluorescence staining of Ki67^+^/CD31^+^ newly proliferated endothelial cells in the ipsilateral hemisphere of mice brain after tMCAO in the PBS and ADSC-EVs groups. The images of the contralateral hemisphere were presented as control. White arrows indicated Ki67^+^/CD31^+^ cells. Scale bar = 20 μm. **H** The enlarged picture showed the Ki67^+^/CD31^+^ cells. **I** Quantifications of Ki67^+^/CD31^+^ cell numbers in the perifocal region at 14 days after tMCAO. *n* = 4 in the PBS and ADSC-EVs groups. Scale bar = 20 μm. Data were mean ± SEM, *p < 0.05, **p < 0.01, ***p < 0.001

To explore the effect of ADSC-EVs treatment on white matter injury after ischemic stroke in mice, we performed immunofluorescence staining of myelin marker MBP at 14 days after tMCAO. Whole brain images and the enlarged images in the striatum showed the MBP intensity and distribution (Fig. 3A). Quantitative analysis showed that the fluorescence intensity and area in the ipsilateral striatum is comparable to the contralateral striatum in the sham group. MPB fluorescence intensity and area dropped in the ipsilateral hemisphere after tMCAO when treated only with PBS. The ratio of MBP fluorescence intensity and area in the ipsilateral striatum to the contralateral striatum was significantly higher in the ADSC-EV group than the PBS group (*p* < 0.05, Fig. 3B, C), indicating that ADSC-EVs treatment reduced white matter damage after tMCAO in mice.

### ADSC-EVs promoted angiogenesis after mouse cerebral ischemia

To assess the effect of ADSC-EVs treatment on angiogenesis in mice after cerebral ischemia, we used CD31 immunofluorescence staining to detect the fluorescence intensity and number of microvessels in the peripheral region of cerebral ischemia at 14 days after tMCAO (Fig. 3D). The average fluorescence intensity of CD31 and the number of microvessels in the ADSC-EV group was higher than the PBS group (*p* < 0.05, Fig. 3E, F). The number of newly proliferated endothelial cells in the ipsilateral hemisphere at 14 days after tMCAO was further detected by CD31/Ki67 double staining (Fig. 3G). The images of contralateral hemisphere of tMCAO mice were presented as control. The vascular morphology of control was thinner than that after ischemic injury and fewer Ki67^+^ signals were detected in the contralateral hemisphere. Therefore, we did not include control group in the quantification. The enlarged image showed the co-localization of CD31 and Ki67 (Fig. 3H). The statistical results showed that the number of newly proliferated endothelial cells in the ipsilateral hemisphere was higher in the ADSC-EV group than that in the PBS group (*p* < 0.01, Fig. 3I).

### ADSC-EVs reversed LPS or OGD induced M1 polarization of cultured microglia

**Fig. 4:**
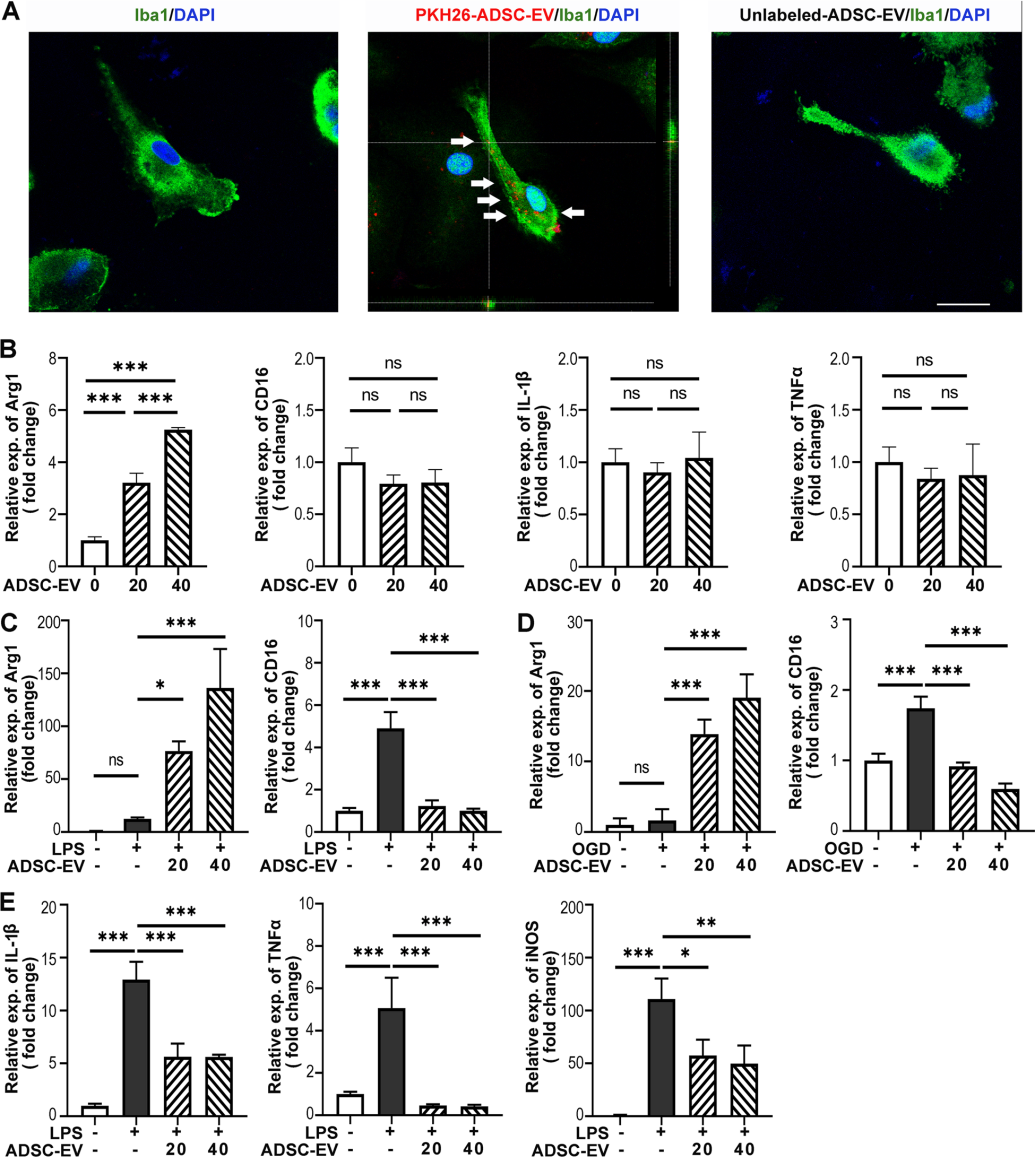
ADSC-EVs shifted microglia polarization to M2 phenotype under LPS or OGD induction. **A** PKH-26 labeled ADSC-EVs (Red) were taken up by Iba1^+^ primary microglia (Green). PKH-26 labeled ADSC-EVs were incubated with primary microglia for 6 h. Scale bar = 10 μm. White arrows indicated ADSC-EVs internalized by cultured microglia. **B** Twenty-four hours after co-incubation with 0, 20, or 40 μg/ml ADSC-EVs, the expression of Arg-1 and proinflammatory marker CD16, IL1-β, TNF-α in primary microglia were detected by qPCR. *n* = 3. **C** Twenty-four hours after 0, 20, or 40 μg/ml ADSC-EVs pretreatment, primary microglia were subjected to 50 ng/ml LPS for 6 h. Then the expression of Arg-1 and CD16 were detected by qPCR. *n* = 3 per group. **D** After 1 h of OGD, primary microglia were subjected to normoxia and glucose synchronously post-treated with 0, 20, or 40 μg/ml ADSC-EVs for 24 h. Then the expression of Arg-1 and CD16 were detected by qPCR. *n* = 6 per group. **E** The expression of inflammatory factors (IL1β, TNF-α and iNOS) under LPS induction were detected by qPCR. *n* = 3 per group. The data were the mean ± SEM, *p < 0.05, **p < 0.01, ***p < 0.001

To further deconvolute the impact of ADSC-EVs on microglia, primary microglia isolated from newborn SD rats was treated with PBS, PKH-26 labeled ADSC-EVs, or unlabeled ADSC-EVs for 6 h (Fig. 4A). The 3D confocal image showed that ADSC-EVs were localized to the cytoplasm of Iba1^+^ microglia in vitro. We next treated the primary microglia with 0, 20, or 40 μg/ml ADSC-EVs for 24 h. qPCR revealed that ADSC-EVs did not affect the expression of M1 marker CD16, IL-1β, and TNF-α; while they up-regulated the expression of M2 marker Arg-1 in a dose-dependent manner (*p* < 0.001, Fig. 4B).

It is well known that LPS induced M1 polarization of microglia [39]. OGD, a common method used to simulate ischemic stroke in vitro, is also known to induce microglia M1 polarization [40]. Therefore, we examined the effects of ADSC-EVs on primary microglial polarization under LPS or OGD conditions. The results showed that ADSC-EVs pretreatment inhibited the LPS-induced up-regulation of CD16 and inflammatory factors Il-1β, TNF-α and iNOS, while increased the expression of Arg-1 in a dose-dependent manner (*p* < 0.05, Fig. 4C, E). Under OGD condition, ADSC-EVs post-treatment also reduced the up-regulation of CD16 and increased the expression of Arg-1 with a dose-dependent manner (*p* < 0.001, Fig. 4D).

### ADSC-EVs shifted microglial polarization to M2 phenotype in tMCAO mice

**Fig. 5:**
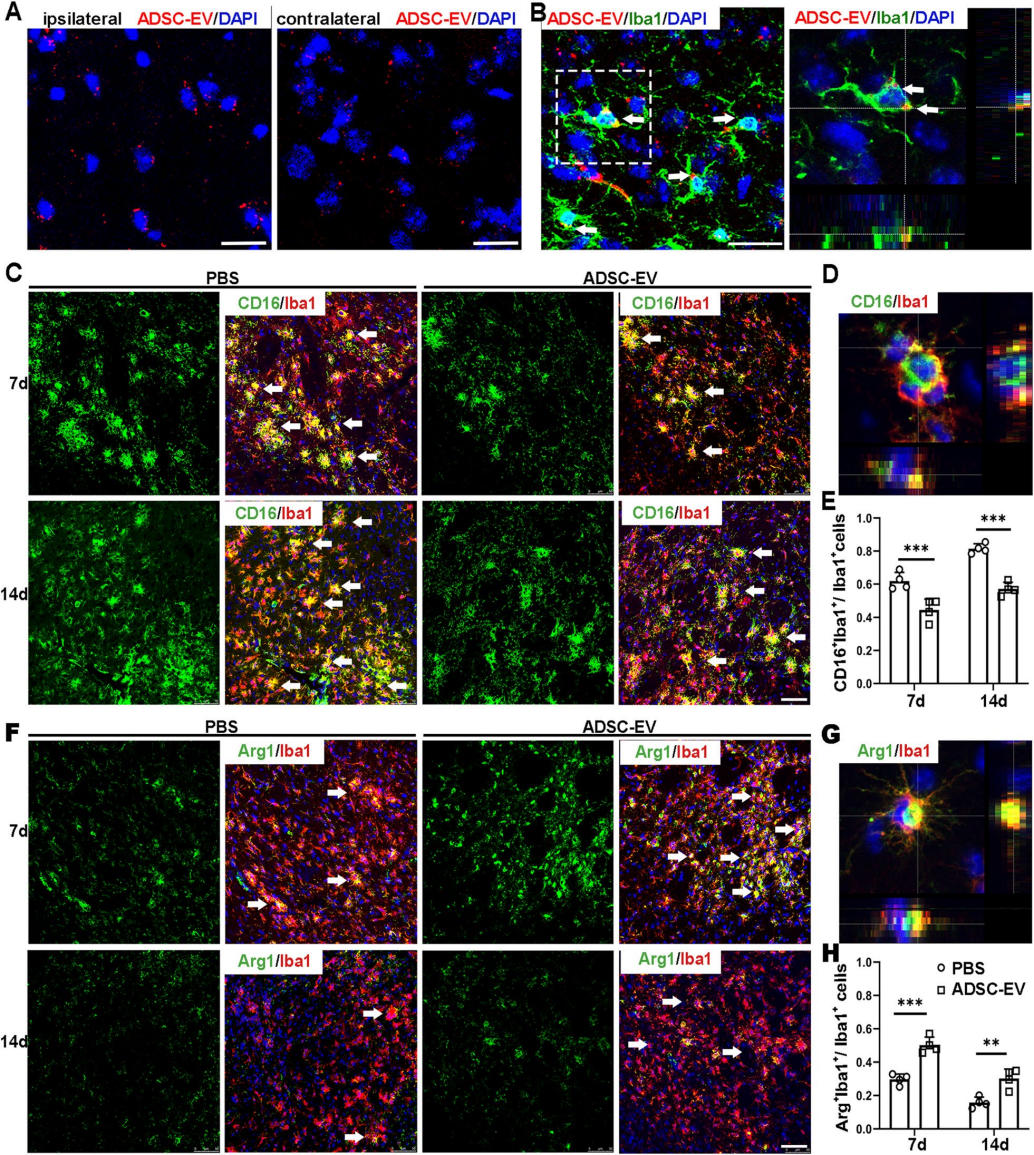
ADSC-EVs shifted the M1/M2 polarization of microglia towards M2 phenotype after tMCAO. **A** PKH-26 labeled ADSC-EVs (Red) were detected around the nucleus (Blue) both in the ipsilateral and contralateral hemisphere of tMCAO mice brain. Scale bar = 15 μm. **B** Immunofluorescence imaging showed the uptake of ADSC-EVs by microglia in vivo. PKH26 labeled ADSC-EVs (Red) were intravenously injected to the mouse at 24 h after tMCAO. The internalization of ADSC-EVs by Iba1^+^ microglia (Green) was detected 1 h after the administration. Scale bar = 25 μm. The 3D image showed the localization of ADSC-EV in the cytoplasm of microglia. White arrows indicated ADSC-EVs internalized by microglia. **C** ADSC-EVs administration reduced the ratio of M1 microglia at 7 days and 14 days after tMCAO. White arrows indicated CD16^+^/Iba1^+^ cells. Scale bar = 50 μm. **D** Zoom in-picture of a single microglia in M1 phenotype. **E** Quantification graph showed the ratio of CD16^+^/Iba1^+^ cells to total Iba1^+^ cells. **F** ADSC-EVs administration increased the ratio of M2 microglia at 7 days and 14 days after tMCAO. White arrows indicated Arg-1^+^/Iba1^+^ cells. Scale bar = 50 μm. **G** Zoom in-picture of a single microglia in M2 phenotype. **H** Quantification graph showed the ratio of Arg-1^+^/Iba1^+^ cells to total Iba1^+^ cells. *n* = 4 per group. Data were mean ± SEM, *p < 0.05, **p < 0.01, ***p < 0.001

Next, we examined the uptake of ADSC-EVs by microglia in tMCAO mice. After 1 h of intravenous injection, PKH26-labeled ADSC-EVs were detected both in the ipsilateral hemisphere and the contralateral hemisphere of the mouse brain (Fig. 5A). Confocal images showed the localization of PKH26-labeled ADSC-EVs in the cytoplasm of Iba1^+^ microglia (Fig. 5B). To detect whether ADSC-EVs could regulate the polarization of microglia after ischemic stroke, we performed CD16/Iba1 and Arg-1/Iba1 double-staining to detect M1 phenotype and M2 phenotype microglia, respectively (Fig. 5C, F). Enlarged views of a single microglia showed the co-localization of Iba1 with CD16 or Arg-1 (Fig. 5D, G). The statistical results showed that the ratio of CD16^+^/Iba1^+^ cells over Iba1^+^ cells at 7 days and 14 days after tMCAO was significantly lower in the ADSC-EV group compared to the PBS group (*p* < 0.001, Fig. 5E). In contrast, the ratio of Arg-1^+^/Iba1^+^ cells over Iba1^+^ cells was higher in the ADSC-EV group than that in the PBS group at both 7 and 14 days after tMCAO (*p* < 0.01, Fig. 5H). The images of contralateral hemisphere were presented in Additional file 1: Fig. S3 and very few polarized microglia were detected in the contralateral hemisphere of tMCAO mice brain. Additionally, the total number of Iba1^+^ cells was not significantly different between the PBS group and the ADSC-EV group both at 7 days and 14 days after tMCAO (Additional file 1: Fig. S4), which was consistent to the study results in a mouse model of traumatic brain injury (TBI) [41].

### miRNA sequencing of ADSC-EVs

**Fig. 6:**
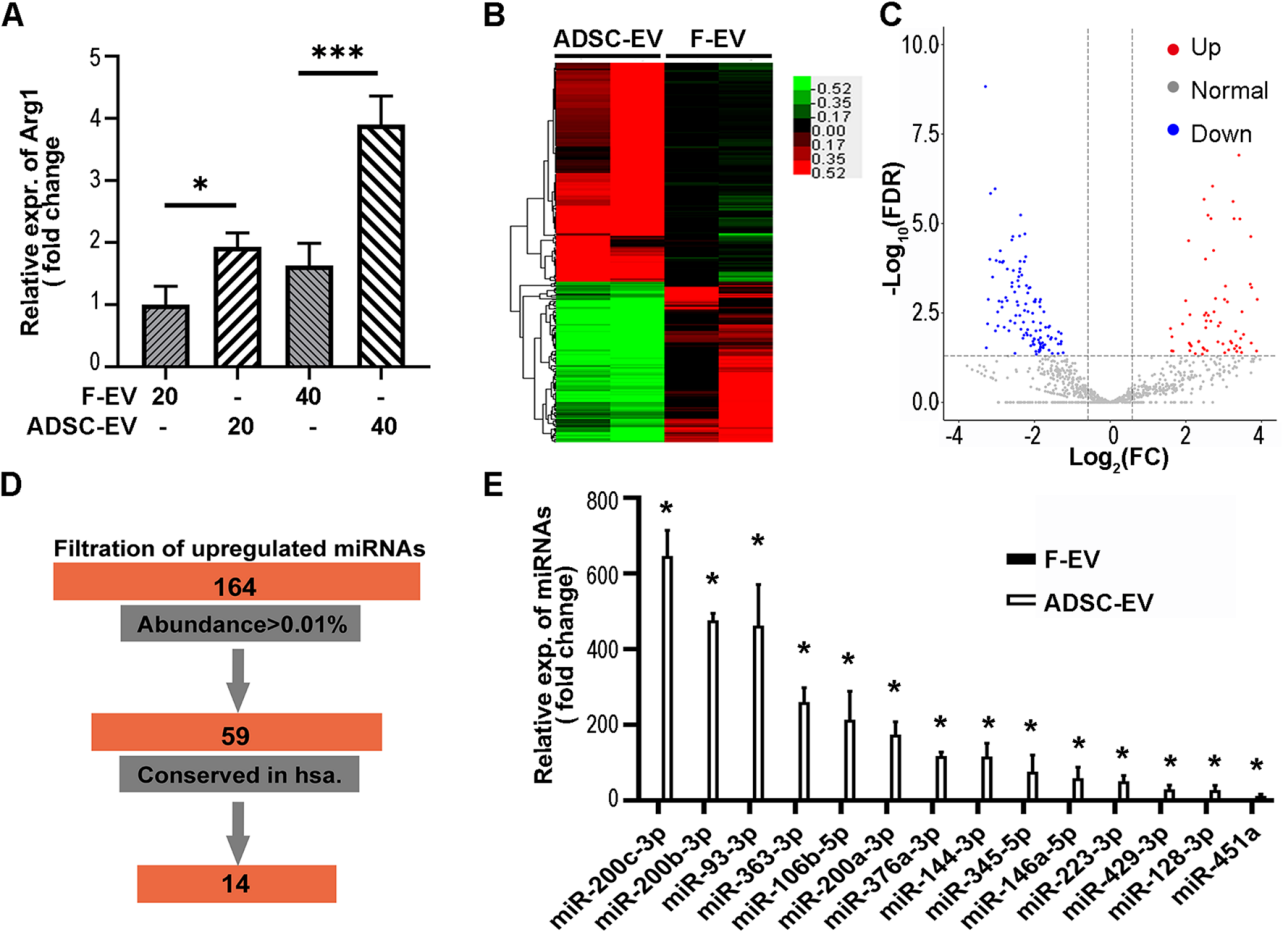
qPCR verified 14 upregulated miRNAs in ADSC-EVs. **A** Twenty-four hours after co-cultivation with fibroblast-EVs or ADSC-EVs, Arg-1 expression in microglia were detected by qPCR. ADSC-EVs were superior than fibroblast-EVs in promoting Arg-1 expression. **B** Heat map showed differently expressed miRNA in ADSC-EVs compared to fibroblast-EVs. **C** Volcano plot revealed 164 upregulated miRNAs and 120 down-regulated miRNAs in ADSC-EVs compared to fibroblast-EVs. **D** 14 up-regulated miRNAs were selected according to abundance more than 0.01% and conservation in human. **E** qPCR verified 14 up-regulated miRNAs. *n* = 3 per group. Data were mean ± SEM, *p < 0.05, **p < 0.01, ***p < 0.001

In previous studies, fibroblast-EVs were used as the control of ADSC-EVs [42, 43]. To compare the difference between fibroblast-EVs and ADSC-EVs in regulating the polarization of microglia, different doses of fibroblast-EVs or ADSC-EVs were used to treat primary microglia cultured in vitro. The results of qPCR revealed that ADSC-EVs up-regulated Arg-1 expression compared to the same dose of fibroblast-EVs (*p* < 0.05, Fig. 6A). The heat map of miRNA sequencing showed the differentially expressed miRNA profiles between fibroblast-EVs and ADSC-EVs (Fig. 6B). The volcano map showed that ADSC-EVs has 164 up-regulated miRNAs and 120 down-regulated miRNAs compared to the fibroblast-EVs (Fig. 6C). We further screened the miRNAs as followings: first, the miRNA should account for at least 0.01% of the total miRNA population in abundance; second, the miRNA should have a conserved counterpart in human. Using these criteria, we identified 14 up-regulated miRNAs in ADSC-EVs (Fig. 6D). Results of qPCR verified that the expression of these 14 miRNAs was significantly up-regulated in ADSC-EVs compared to fibroblast-EVs (*p* < 0.05, Fig. 6E).

### ADSC-EVs regulated microglial polarization through inhibiting STAT1 and PTEN

**Fig. 7:**
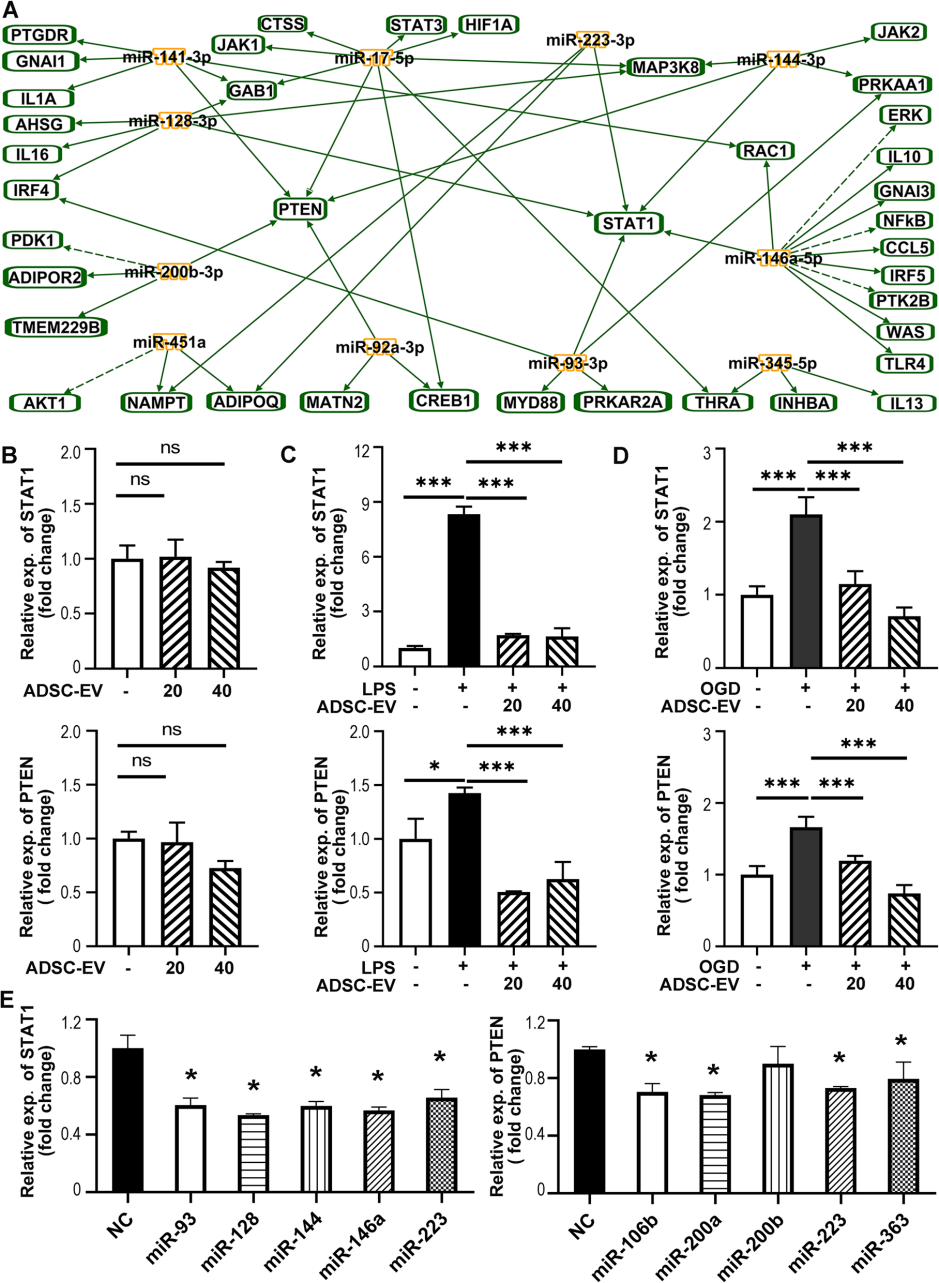
ADSC-EVs inhibited LPS or OGD induced up-regulation of STAT1 and PTEN. **A** The relationship between 14 up-regulated miRNAs and proteins related to microglia polarization were analyzed by IPA, and STAT1 and PTEN were highlighted as the targets of these miRNAs. **B** qPCR revealed that ADSC-EVs administration did not significantly change STAT1 and PTEN expression in primary microglia under normal condition. **C** ADSC-EVs pretreatment attenuated LPS induced up-regulation of STAT1 and PTEN. **D** ADSC-EVs post-treatment significantly dampened the OGD-induced upregulation of STAT1 and PTEN. **E** qPCR confirmed that STAT1 expression was down-regulated after the transfection of miR-93-3p mimics, miR-128-3p mimics, miR-144-3p mimics, miR-146a-5p mimics, and miR-223-3p mimics compared with the negative control in BV_2_ microglia. The expression of PTEN was down-regulated after the transfection of miR-106b-5p mimics, miR-200a-3p mimics, miR-223-3p mimics, and miR-363-3p mimics. *n* = 3 per group. Data were mean ± SEM of three independent experiments, *p < 0.05, **p < 0.01, ***p < 0.001

To explore the underlying mechanism of ADSC-EVs mediated microglial polarization, we first found out 85 proteins related to microglia or macrophage polarization from previous studies and IPA system. Then the relationships between 14 upregulated miRNAs in ADSC-EVs and 85 related proteins were analyzed in IPA system. The results showed that 13 of the 14 upregulated miRNAs were involved in microglial polarization. Notably, STAT1 and PTEN were highlighted as two downstream proteins targeted by most of these miRNAs (Fig. 7A). Transcription factor STAT1 is closely related to both microglia M1 polarization and the expression of inflammatory cytokines IL-1α, IL-1β, TNF-α [44]. Inhibition of PTEN has also been demonstrated to promote the polarization of microglia to M2 phenotype [45]. We further examined the effects of ADSC-EVs treatment on STAT1 and PTEN expression in cultured microglia. The results showed that ADSC-EVs treatment did not affect STAT1 or PTEN expression under normal culture condition (Fig. 7B), while pre-treatment or post-treatment of ADSC-EVs significantly inhibited LPS or OGD-induced upregulation of STAT1 and PTEN (*p* < 0.001, Fig. 7C, D). To confirm the causal relationships between miRNAs and microglial polarization-related proteins, we transfected BV_2_ microglia with 100 nM negative control, or 100 nM mimics of each of the upregulated miRNA. The results confirmed that miR-93-3p mimics, miR-128-3p mimics, miR-144-3p mimics, miR-146a-5p mimics, and miR-223-3p mimics decreased the expression of STAT1, and miR-106b-5p mimics, miR-200a-3p mimics, miR-223-3p mimics and miR-363-3p mimics decreased the expression of PTEN (*p* < 0.05, Fig. 7E).

## Discussion

Microglia are the central integrators in neurological diseases, as well as vital mediators of the CNS during developmental and pathological progressions [46]. Interactions between microglia and neurons, astrocytes, oligodendrocytes, and other cells in the CNS determine the pathological outcomes of brain diseases [47]. After ischemic stroke, microglia can either aggravate injury or facilitate repair [48, 49]. Classically activated M1 microglia release proinflammatory cytokines and destroy adjacent neurons and oligodendrocytes. Conversely, pro-regenerative M2 microglia secrete anti-inflammatory mediators and express Arg-1 instead of iNOS [47]. Recent studies have argued that the simple division of microglia into M1 and M2 phenotype is too rough to reflect the complex and dynamic state of microglia under different disease conditions, because overlapping phenotypes and intermediate stage also exist in different subpopulations of microglia [50, 51]. Despite the controversy on microglia phenotyping, strategies aimed to redirect microglia from detrimental to beneficial functions is a potential approach to target microglia therapeutically [52]. Based on these considerations, we chose to study the role of microglia in ischemic stroke with the broad concepts of M1 and M2 phenotype instead of defining each subpopulation further.

ADSCs have been considered as the optimal source of MSCs with tremendous potential in the treatment of cerebral ischemia. Adipose stem cells and their derived derivatives have been demonstrated to be effective in regulating macrophage polarization [25, 53]. The activation of microglia is similar to those of peripheral macrophages after ischemic stroke. In this study, we investigate whether the polarization of microglia was regulated by ADSC-EVs after tMCAO. Our results indicate that ADSC-EVs up-regulate Arg-1 expression and inhibit CD16 expression both in vivo after tMCAO and in vitro under LPS/OGD condition. As aforementioned, the shifting of microglial polarization could be beneficial to various cell types in the CNS under stress. Here, we detect an increase in angiogenesis and a decrease in MBP loss at 14 days after cerebral ischemia in the ADSC-EVs treated group compared to the PBS control. It is well established that M2 microglia promote angiogenesis [54, 55]. For instance, culture supernatant of M2 microglia promotes angiogenesis in vitro in human brain microvassal endothelial cells (HBMECs) [50]. M2 microglia also protect against white manner injury and drive oligodendrocyte differentiation during CNS remyelination [11, 56]. These evidences suggest that the increased angiogenesis and decreased demyelination are at least partially associated to the shifted microglial polarization by ADSC-EVs. The improvement of neurological recovery and substantial reduction of brain atrophy after ischemic stroke could be attributed to the beneficial interactions between M2 microglia and different cell types in the CNS. It should be noted that Iba1^+^ cells in the ischemic brain represent both microglia and macrophages. Since peripherally infiltrated macrophages constitute only a small percentage of the total population of Iba1^+^ cells (less than 5%), we do not aim to distinguish between the resident microglia and circulating macrophages infiltrated to the injured brain in this study [57]. Systematically administrated ADSC-EVs were soon internalized by the recipient cells [12]. Considering for their short half-life, we did not record the EVs fate in mice brain for a longer time. However, it was still a meaningful topic to track and increase the distribution of EVs in mice brain after intravenous injection. Apart from microglia, they could also be taken up by other cells beside microglia and improve the cerebral ischemic injury subsequently. Since the contribution of other cell types could not be excluded here, such as neurons, astrocytes, OPC, or peripheral immune cells, we concluded that ADSC-EVs exert therapeutic function after cerebral ischemia partly through direct delivery cargos to microglia and regulating their polarization.

Under normal condition, ADSC-EVs do not affect the baseline expression of M1 microglia marker and inflammatory cytokines in rat primary microglia. Considering for the limited number of microglia that could be isolated from postnatal ICR mouse as well as the difference between BV_2_ cell line and primary microglia [58], we isolated primary microglia from postnatal SD rats to explore the role of ADSC-EVs derived from ICR mice in vitro. In contrast, under LPS/OGD stress, ADSC-EVs inhibit the M1 microglial polarization and the expression of pro-inflammatory cytokines IL-1β, TNF-α and iNOS. Meanwhile, Arg-1 expression is up-regulated by ADSC-EVs in a dose-dependent manner. These results suggest that the contents in ADSC-EVs contribute to the promotion of microglia M2 polarization.

miRNAs are main functional cargos in EVs and have been demonstrated to participate in various physiological and pathological processes [59]. In this study, we perform miRNA sequencing of ADSC-EVs using fibroblast-EVs as a control and identified 284 differentially expressed miRNAs, of which 164 are up-regulated in ADSC-EVs. Fibroblast have regularly been used as the control cell for MSCs in previous studies [60], and fibroblast-EVs have been used as the control for ADSC-EVs [42, 43]. The results of IPA analysis highlight STAT1 and PTEN as two downstream proteins targeted by most up-regulated miRNAs in ADSC-EVs. It is predicted that 13 among the 14 up-regulated miRNAs could negatively regulate one or more proteins related to microglial polarization. Among these 13 miRNAs, miR-93-3p, miR-128-3p, miR-144-3p, miR-146a-5p, and miR-223-3p negatively regulate STAT1, and miR-106b-5p, miR-200a-3p, miR-200b-3p, miR-223-3p, and miR-363-3p negatively regulate PTEN. STAT1 is a transcription factor that plays a critical role in microglia pro-inflammatory pathway and closely related to the expression of pro-inflammatory cytokines such as IL-1α, IL-1β, TNF-α [61, 62]. STAT1 and NFκB are main responding transcription factors in both LPS and IFNγ mediated inflammatory signaling in microglia [39, 63]. SOCS1 and SOCS3 recruited by IL-10 suppress the JAK/STAT-1 signaling to inhibit pro-inflammatory polarization [62]. Inhibiting STAT1 activation and up-regulation can reduce the M1 microglia/macrophage polarization [44, 64]. PTEN convert PIP3, the substrate of Akt activation, to PIP2. Thus, inhibition of PTEN results in activation of Akt, which can further shift microglia from the detrimental M1 phenotype toward the protective M2 phenotype and thereby exert the protection of neighboring oligodendrocytes [45]. PTEN knockout promotes M2 microglial polarization and inhibit the pro-inflammatory response after ischemic brain injury [65]. These results indicate that both STAT1 and PTEN are involved in the regulation of microglial polarization and inhibition of STAT1 or PTEN contribute to increase M2 phenotype and decrease M1 phenotype of microglia. Further study confirms that ADSC-EVs could inhibit the up-regulation of STAT1 and PTEN in primary microglia under LPS/OGD conditions. In addition, mimics of these miRNAs decrease the expression level of STAT1 or PTEN after transfected into BV_2_ microglia, except for miR-200b-3p mimics. This result is not surprising given the low score of binding sites between STAT1 and miR-200b-3p predicted by TargetScan. Therefore, we speculate that ADSC-EVs exert a regulatory function on microglial polarization by direct delivery of miRNA cargos to recipient microglia. We did not focus on only one miRNA to study its mechanism further, because we believe that the regulation effect of ADSC-EVs on microglia polarization is a result of a network interaction in which multiple miRNAs participated.

## Conclusion

Collectively, the present study has investigated the efficacy of ADSC-EVs in treating ischemic brain injury using a mouse model and explored the mechanisms by which ADSC-EVs exert their function. Our results demonstrated that ADSC-EVs promoted M2 polarization of microglia both in vivo and in vitro. In addition, we identified STAT1 and PTEN as two downstream targets of upregulated miRNAs in ADSC-EVs and confirmed their inhibition by ADSC-EVs in cultured microglia. These results supported that ADSC-EVs could regulate microglial polarization, which was at least partly attributed to the delivery of miRNAs to inhibit the expression of STAT1 and PTEN. Our results provided a potential therapeutic target for ischemic brain injury.

## Supplementary Information

**Additional file 1: Figure S1.** Cell morphology and differentiation of ADSCs. **Figure S2.** Laser speckle images of tMCAO mice brain. **Figure S3.** Very few polarized microglia were detected in the contralateral hemisphere of tMCAO mice. **Figure S4.** ADSC-EVs administration did not decrease the population of Iba1^+^ cells in the ipsilateral hemisphere of tMCAO mice.

## Data Availability

The datasets supporting the conclusions of this article are included within the article and its additional files.

## Abbreviations

ADSC: Adipose-derived stem cells
Arg-1: Arginase-1
CD16: Cluster of differentiation marker 16
CBF: Cerebral blood flow
CNS: Central nervous system
EV: Extracellular vesicles
F-EV: Fibroblast derived extracellular vesicles
HBMECs: Human brain microvassal endothelial cells
IL-1β: Interleukin-1β
iNOS: Inducible nitric oxide synthase
IPA: Ingenuity pathway analysis
LPS: Lipopolysaccharides
MBP: Myelinbasicprotein
miRNA: MicroRNA
mNSS: Modified neurological severity score
MSCs: Mesenchymal stem cells
NC: Negative control
NTA: Nanoparticle tracking analysis
OGD: Oxygen–glucose deprivation
OPC: Oligodendrocyte progenitor cells
PTEN: Phosphatase and tensin homolog deleted on chromosome ten
STAT1: Signal transducers and activators of transcription 1
TBI: Traumatic brain injury
TEM: Transmission electron microscope
tMCAO: Transient middle cerebral artery occlusion
TNF-α: Tumor necrosis factor α
TSG101: Tumor susceptibility gene 101
WAT: White fat tissue
