# Supplementary material for: Extracellular vesicles from adipose-derived stem cells promote microglia M2 polarization and neurological recovery in a mouse model of transient middle cerebral artery occlusion
Source: Stem Cell Res Ther. 2022 Jan 20;13:21. doi: 10.1186/s13287-021-02668-0 (PMC8772170; doi:10.1186/s13287-021-02668-0)
Supplement: Supplementary file 1 — Additional file 1: Figure S1. Cell morphology and differentiation of ADSCs. Figure S2. Laser speckle images of tMCAO mice brain. Figure S3. Very few polarized microglia were detected in the contralateral hemisphere of tMCAO mice. Figure S4. ADSC-EVs administration did not decrease the population of Iba1+ cells in the ipsilateral hemisphere of tMCAO mice. [file 13287_2021_2668_MOESM1_ESM.docx]

**Supplementary Materials**

Fig S1: Cell morphology and differentiation of ADSCs.

Fig S2: Laser speckle images of tMCAO mice brain.

Fig S3: Very few polarized microglia were detected in the contralateral hemisphere of tMCAO mice.

Fig S4: ADSC-EVs administration did not decrease the population of Iba1^+^ cells in the ipsilateral hemisphere of tMCAO mice.


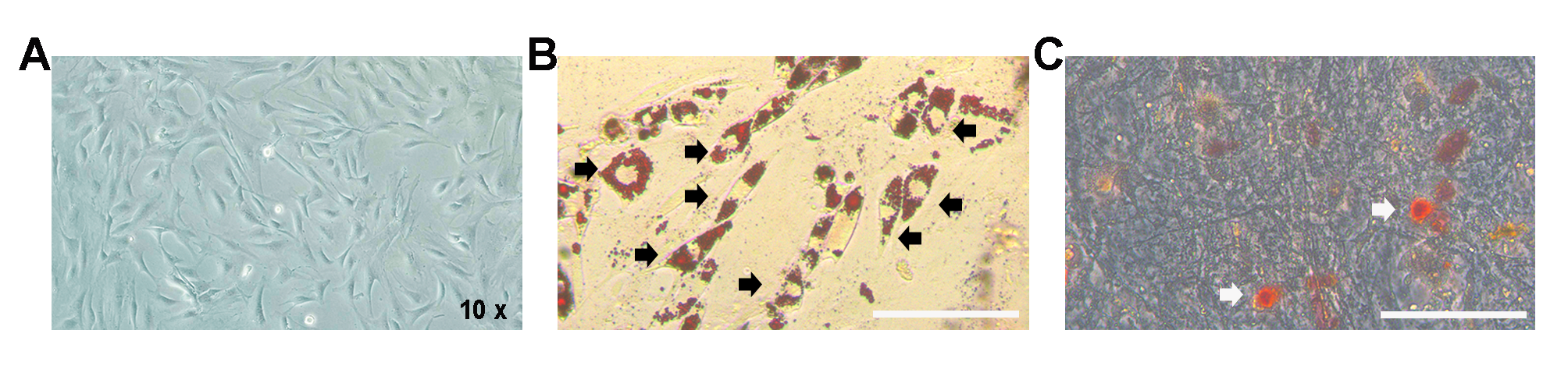


**Supplementary Figure 1:** **Cell morphology and differentiation of ADSCs.**

(A)The morphology of ADSCs. 10x. (B) Oil Red O staining showed that a large number of red oil droplets gathered in the cytoplasm of ADSCs after adipogenic induction for 7 days. Black arrows indicated adipocytes. Scale bar=100 μm. (C)Alizarin Red S staining showed red calcium nodules after osteogenic induction for 14 days. White arrows indicated calcium deposition. Scale bar=100 μm.


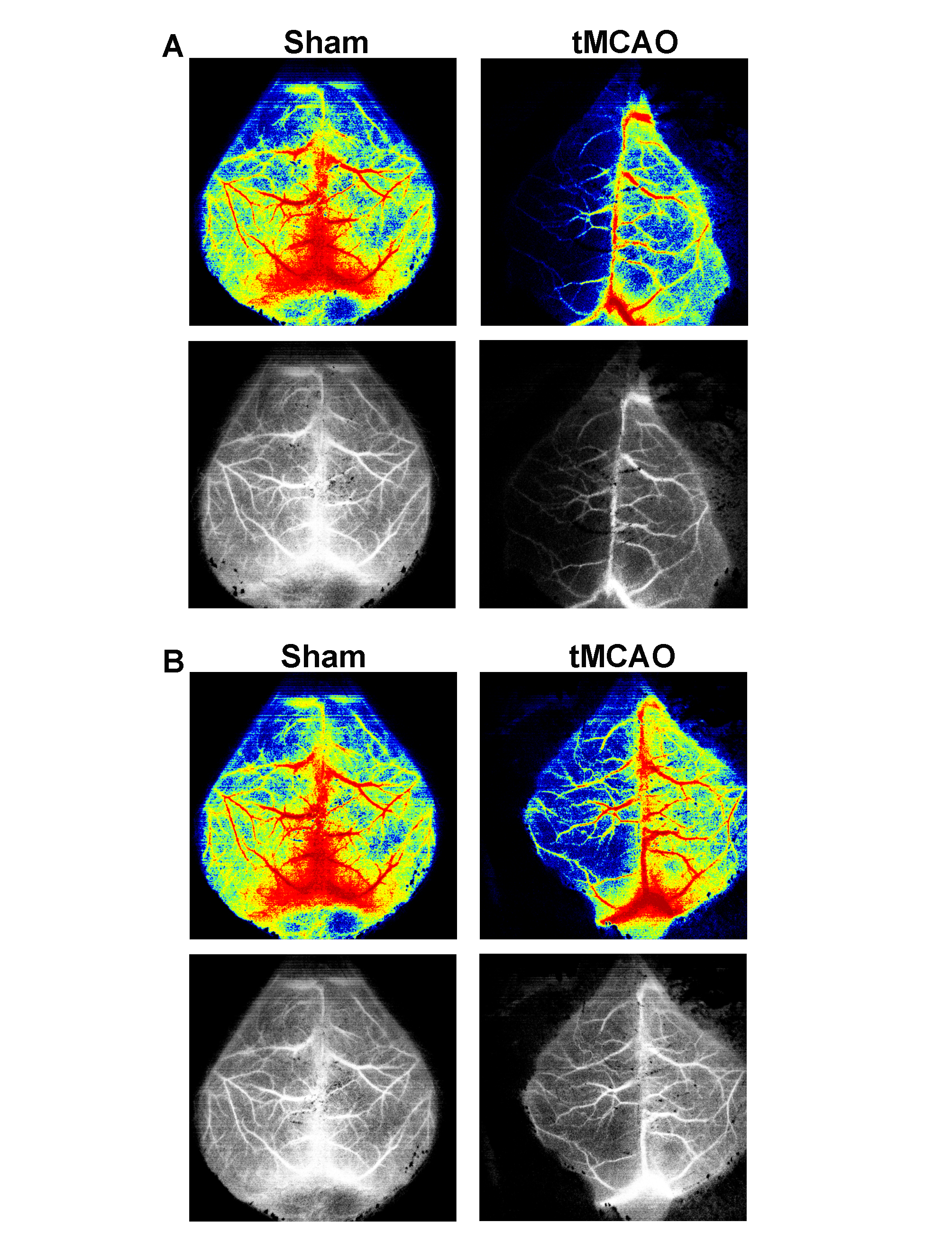


**Supplementary Figure 2:** **Laser speckle imagines of tMCAO mice brain.**

(A) Cerebral blood flow of mice during MCA occlusion in Sham group or tMCAO group. (B) Cerebral blood flow of mice after MCA reperfusion in Sham group or tMCAO group.


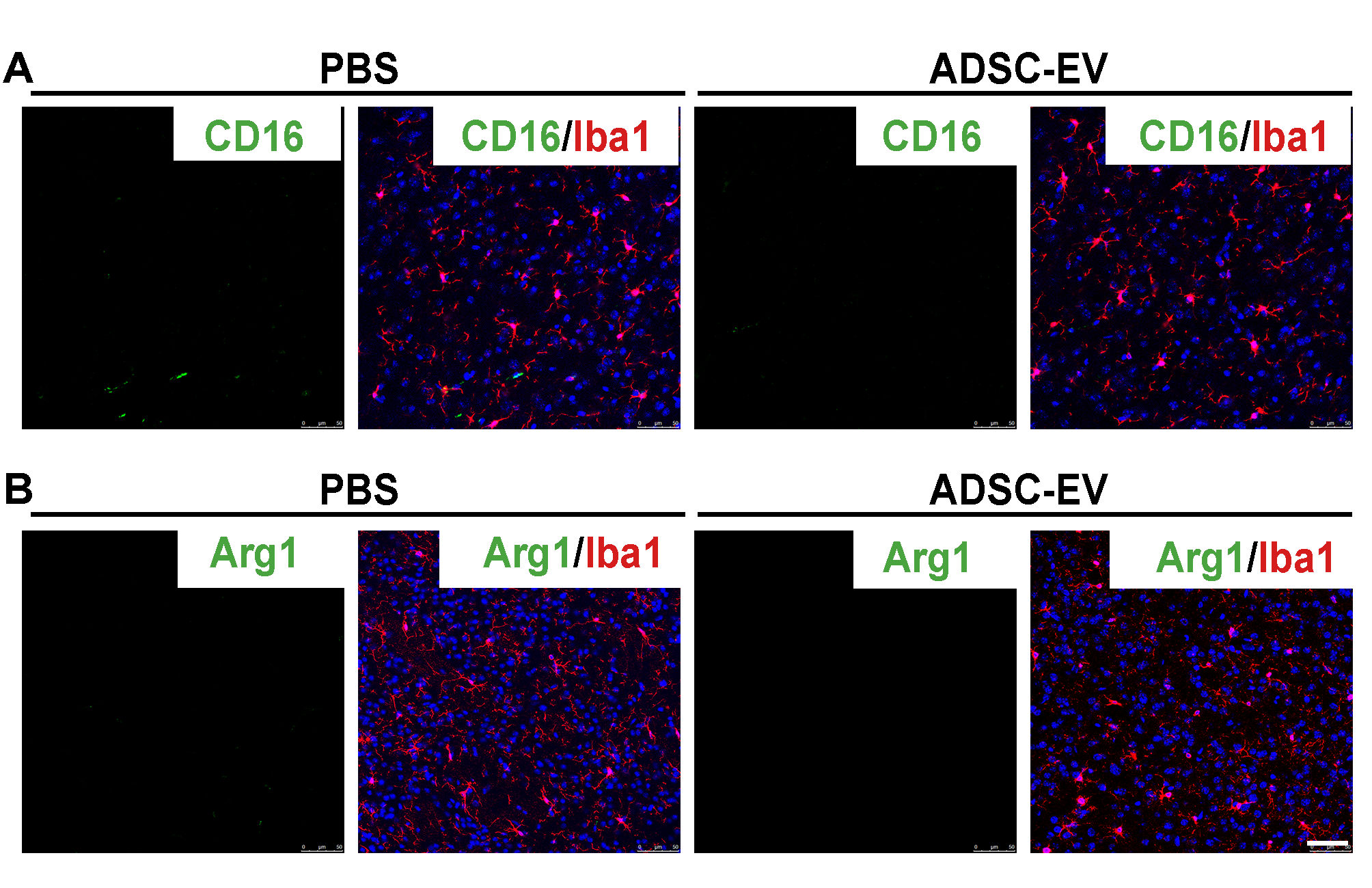


**Supplementary Figure 3:** **Very few polarized microglia were detected in the contralateral hemisphere of tMCAO mice.**

(A) Very few CD16^+^/Iba1^+^ cells were detected in the contralateral hemisphere of tMCAO mice both in PBS and ADSC-EV group. (B) Very few Arg-1^+^/Iba1^+^ cells were detected in the contralateral hemisphere of tMCAO mice both in PBS and ADSC-EV group. Scale Bar=50 μm.


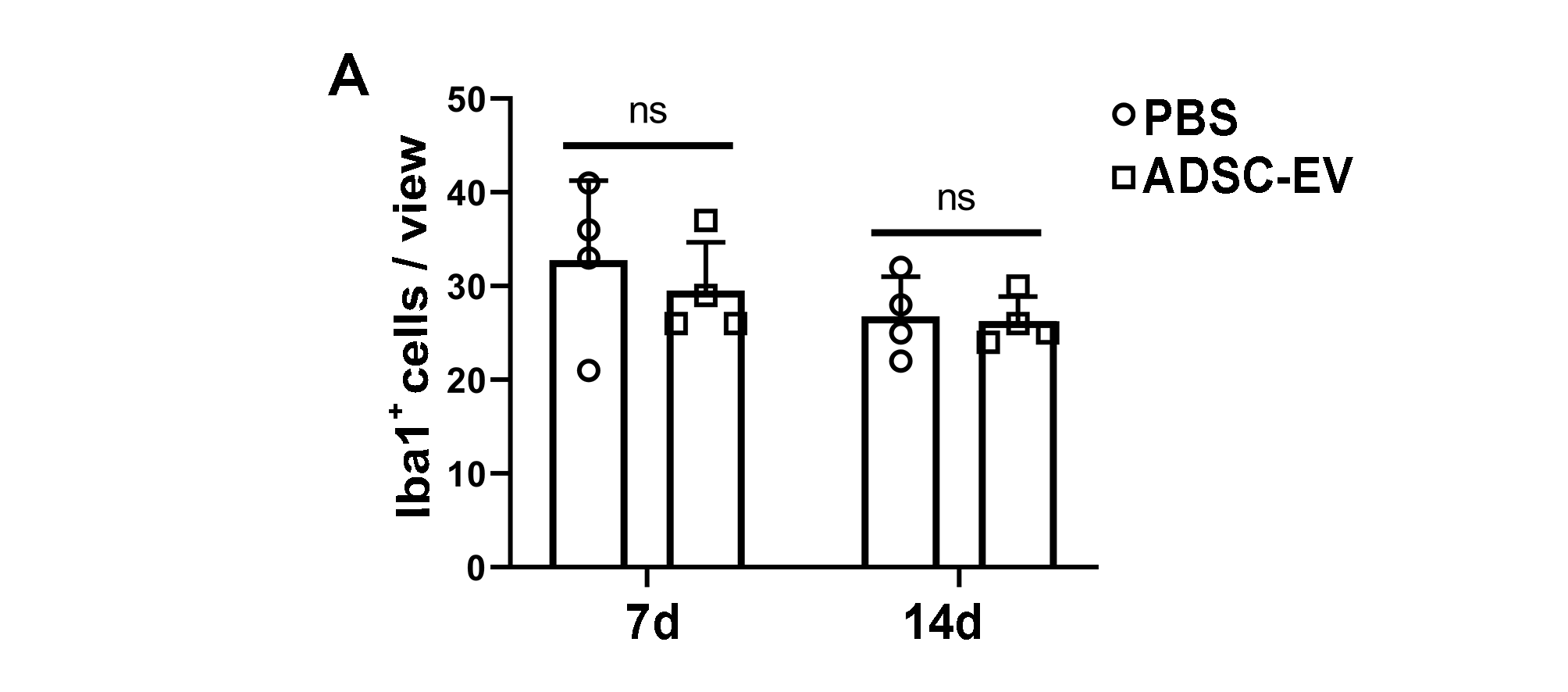


**Supplementary Figure 4:** **ADSC-EVs administration did not decrease the population of Iba1^+^ cells in the ipsilateral hemisphere of tMCAO mice.**

(A) Quantification of the total number of Iba1^+^ cells showed no difference between the PBS and ADSC-EV group both at 7 and 14 days after tMCAO.
